# Corrosion Protection and Heat Resistance of Paints for Outdoor Use

**DOI:** 10.3390/ma16072753

**Published:** 2023-03-29

**Authors:** Ilona Felhősi, Lívia Molnárné Nagy, Szilvia Horváth, Tamás Pozman, János Bognár, Tamás Szabó, Zsófia Keresztes

**Affiliations:** 1 Functional Interfaces Research Group, Institute of Materials and Environmental Chemistry, Research Centre for Natural Sciences, Magyar Tudósok krt. 2, 1117 Budapest, Hungary; 2 Industrial Paint Research Ltd., Venyige u. 3, 1108 Budapest, Hungary

**Keywords:** heat-resistant paints, corrosion-resistant paints, polysiloxane coating, zinc phosphate pigment, ethyl silicate coating, zinc-rich primers

## Abstract

Innovative heat- and corrosion-resistant coating approaches, applicable in indirect-food-contact outdoor environments, have been developed. Two systems, a direct-to-metal single-layer, polysiloxane-based, oven-dried system and a bilayer, zinc phosphate active pigment-containing, ambient-cured system were developed to overcome the shortcomings of the traditional bilayer, zinc-rich primer-based heat-resistant surface-protective solutions for outdoor cooking equipment, such as barbecue grills. This case study aims to optimize the application conditions, measure and evaluate the impact of surface preparation and compare thermo-resistant and anticorrosive properties of different coating systems focusing on eco-efficiency. The anticorrosion efficiency of the coatings was characterized using salt-spray chamber corrosion tests and electrochemical impedance spectroscopy. The thermo-resistant character of the coatings was tested by cyclic and constant heat treatment, after which the physical integrity of the coatings was evaluated by optical microscopy. In the overall performance of the coatings, the roughening of the steel substrate surface and the thickness of the coatings were also considered as influential parameters. The study revealed that the newly developed coatings have superior anticorrosion performance to the usually applied Zn-rich coating. The Single-layered Coating has excellent corrosion resistance under certain conditions and has the advantage of fast layer application. The Bilayered Coating showed excellent heat- and corrosion-resistance properties even on a surface without sand-blasting.

## 1. Introduction

Protection of outdoor carbon steel equipment with decorative coatings (e.g., exhausters, BBQs chimneys, furnaces, ovens, grills) is a challenging task. For outdoor applications, the coating must also comply with proper function and different weather conditions in addition to aesthetics; in other words, the applied coatings are expected to be both heat- and corrosion-resistant. Furthermore, coatings for cooking devices must meet indirect food safety regulations, too.

High-temperature coatings are designed to maintain barrier performance at temperatures above environmental conditions. Silicon-based coating materials receive special attention in the field of protective coatings for high-temperature applications due to their good thermal stability properties [1,2,3]. The binder that is commonly used in coatings for high-temperature applications is polysiloxane [4]. The polymer backbone is usually functionalized with aliphatic and aromatic groups to improve thermal stability, and it has been investigated for its high-temperature applications [5,6,7,8,9,10].

Zinc-rich (zinc powder-containing) coatings offer outstanding cathodic protection to steel substrates, because, after curing of the coating, zinc provides the matrix with electrical conductivity. It is established that in earlier stages of defensive coating performance, there is a period of electrochemical activity, in which the preferential attack of the corrosive medium on zinc particles ensures cathodic protection for steel substrate [11,12].

Zinc-rich inorganic coatings based on ethyl silicate binder, with the combination of prominent characteristics of both components, are classified among high-performance coatings, used for the protection of steel against corrosion under severe conditions such as underground environment, marine or industrial atmosphere and nuclear power plants. These coatings provide very efficient corrosion protection to steel substrates exposed to high temperatures up to 400 °C [13,14].

One of the most established environmentally acceptable anticorrosive pigments is zinc phosphate Zn_3_(PO_4_)_2_.2H_2_O, widely used in corrosion-resistant coatings on metal surfaces as a primer pigment [15,16,17]. It has become one of the most used corrosion inhibitors. The corrosion protection effect of zinc phosphate is based on the formation of the protective iron phosphate film. When the corrosive medium permeates the coating, the zinc phosphate partly dissolves [16], and a protective film is formed that contains carboxyl and hydroxyl groups, which can enhance the adhesion between the coating and the substrate [17].

There is a wide variety of heat-resistant coatings and coatings for corrosion protection, but limited numbers of studies deal with the combination of these two criteria [18,19,20,21]. Given the current state of the art, developing a coating with high mechanical and chemical resistance, high flexibility, good crack-free adhesion and long-term heat resistance using liquid silicone resin-based paints has been an unresolved problem, especially for non-sand-blasted steel surfaces. A formulation developed by Industrial Paint Research Ltd. gives a coating with higher hardness and scratch resistance, with good adhesion on steel plates even after sustained high-temperature heat exposure of 400–600 °C, and without the brittleness phenomenon known from heat-resistant two-component paints. A range of paints was developed and tested with reduced carbon content and reduced volatile organic compound (VOC) emissions, which meet high quality requirements and provide an aesthetic coating. In addition to environmental and sustainability considerations, a range of liquid paints that are easy to apply and have outstanding protective properties ensuring that coated objects retain their function and aesthetic properties over time was developed.

The aim of the present study is the characterization and comparison of the two above-mentioned innovative coating systems in terms of anticorrosive and thermo-resistant characteristics. One coating is a single-layer approach, a heat-resistant, solvent-based, zinc phosphate-containing paint bearing significant barrier properties (Single-Layer Coating), and the other is an ambient curing bilayer coating system which consists of a Zn phosphate-containing primer and a barrier topcoat (Bilayered Coating II). Both coating systems are proven to be suitable for outdoor grill equipment constructed from steel. In the present study, salt-spray tests and electrochemical impedance spectroscopy (EIS) measurements were used to monitor the corrosion, and optical microscopy was used to test the physical integrity of the coatings.

Key parameters affecting the corrosion protection efficiency of the heat-resistant paint coating were examined to optimize the application conditions: (i) The protective capacity of the coating as a function of the layer thickness was investigated. (ii) The influence of continuous and cyclic heat loads on the corrosion resistance of the paint coatings is presented. (iii) The effect of the steel type on the protective effect of the paint coatings is also important; therefore, comparative tests were also performed on cold-rolled and hot-rolled steel sheets. (iv) Anticorrosive properties of the investigated coating systems were compared on sand-blasted and smooth, non-sand-blasted substrate steel surfaces.

The protective effect of both coating systems is compared with a traditional bilayer coating system recommended for high-temperature use in fireplaces, consisting of a zinc powder-containing ethyl silicate primer and a barrier topcoat (Bilayered Coating I).

## 2. Materials and Methods

### 2.1. Paint Coating Systems

*Carbon steel substrates*: Cold-rolled steel: DC01 (composition: C% ≤ 0.12; P% ≤ 0.045; S% ≤ 0.045; Mn% ≤ 0.60; Ti% ≤ 0.008; Al% ≥ 0.02), sheet thickness: 1 mm. Hot-rolled steel: S235 (composition: C% ≤ 0.17; Si% ≤ 0.3; Mn% ≤ 1.4; P% ≤ 0.035; S% ≤ 0.035; Cu% ≤ 0.55; N% ≤ 0.012), sheet thickness: 3 mm. Sandblasting and roughening were performed with EKF80 (electrocorundum white)-type grains 0–14 days before application, optimally within 2 days before painting.

*Single-layered Coating:* Single-layered polysiloxane-based heat-resistant coating formulated with active zinc phosphate (Stoving, ZnP) pigments (2K HEAT MIO) was applied at 60–100 μm DFT on carbon steel. The coating was sprayed on a smooth, degreased, cold-rolled plate, as well as on a sand-blasted plate. Oven-baking: at 220 °C for 40 min

*Bilayered Coating I*: As a reference, this bilayer coating system consisting of ethyl silicate-based zinc-rich primer and a barrier topcoat (2K Thermoresist HSR ECO) was applied at 120 μm DFT. The primer coating was only applied to a sand-blasted plate, as it has no proper adhesion on a smooth surface. The topcoat was sprayed and air-dried. Drying: at 80 °C for 30 min.

**Figure 1:**
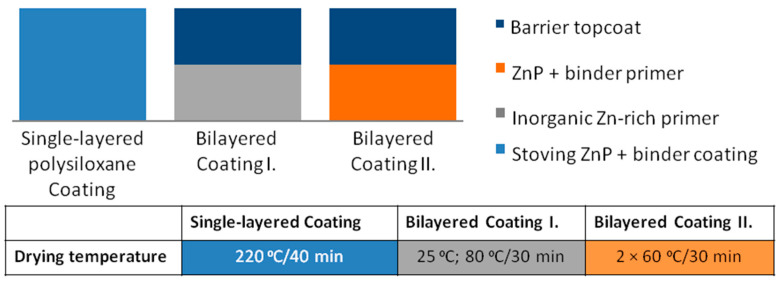
Summary of the investigated paint coating systems.

*Bilayered Coating II*: 2K Thermoresist HSR-SG primer + 2K Thermoresist HSR ECO black two-layer coating system was applied at different thicknesses (50–120 µm) on a carbon steel substrate. The primer coating (2K Thermoresist HSR-SG primer) containing Zn phosphate pigment was sprayed onto the appropriate plate and then allowed to dry at room temperature for 20 min, and it was forced-dried at 60 °C for 30 min. The topcoat (2K Thermoresist HSR ECO black) was sprayed and air-dried at 60 °C for 30 min. The investigated paint coating systems are summarized in Figure 1.

### 2.2. Heat Treatment of Painted Steel Sheets

Heat treatment of coated steel sheets was performed in two different heat profiles:

- Continuous method: holding panels at 300 °C for 15 h and cooling to room temperature.
- Cyclic method: holding panels at 300 °C for 3 h and subsequent cooling to room temperature, with a repeat number of 5 (total 5 × 3 h).

### 2.3. Salt-Spray Test

The temperature in the device was set to 35 ± 2 °C. The concentration of the NaCl solution was 5%. The solution was prepared with distilled water, and its pH was adjusted between 6.5 and 7.2 using a hydrochloric acid solution or sodium bicarbonate. The salt solution was sprayed into the test space using compressed air through a nozzle. The pulverization was set so that 1–2.5 mL/hour of salt solution reached a surface of 80 cm^2^. The sample plates were placed in a plastic sample holder in the testing room in such a way that the tested surface formed an angle of 15–25° to vertical.

### 2.4. Optical Microscopy

Microscopic images were taken with a Zeiss Axio Imager A1 (Carl Zeiss AG, Göttingen, Germany).

### 2.5. Electrochemical Impedance Spectroscopy (EIS) Measurements

**Figure 2:**
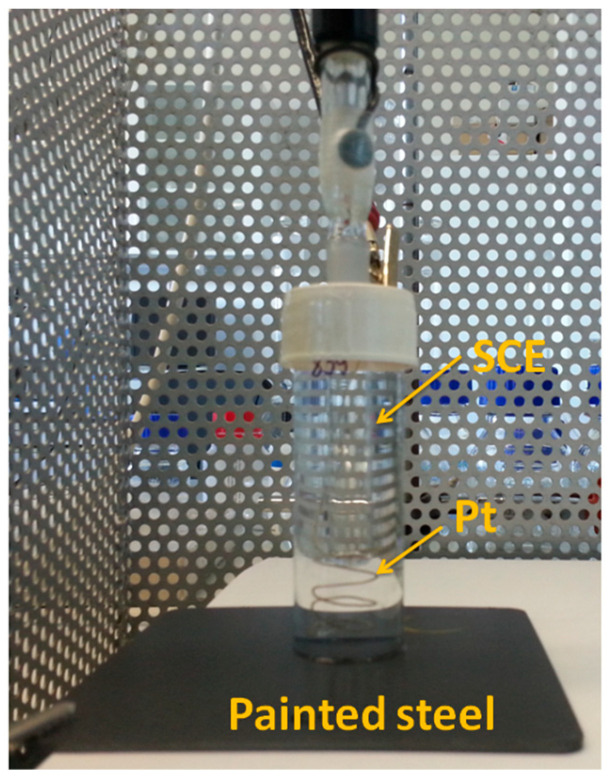
Picture of the electrochemical cell setup.

EIS measurements were performed using a Solartron 1286 potentiostat (“electrochemical interface”) in conjunction with a Solartron 1250 frequency response analyzer (Solartron Instruments, Farnborough, England). Instrument control and the fitting of the measured impedance spectra were performed using CorrWare/Corview version 3.5f and ZPlot/Zview version 3.5i software (Scribner Associates, Inc., Southern Pines, NC, USA). The electrochemical cell was a three-electrode cell with a saturated calomel electrode (SCE) as the reference electrode, a platinum wire as a counter electrode and the paint-coated sample as a working electrode. The body of the cell was a glass tube (Ø 22 mm), which was mounted to the painted steel with epoxy glue (Figure 2). Measurements were performed in 20 mL 5% NaCl solution at room temperature. In order to monitor the time dependence of corrosion, we compiled a series of measurements in which the open-circuit potential (OCP) and EIS measurements were repeated cyclically. Impedance spectra were recorded at OCP using a 10 mV effective amplitude sinusoidal signal with 10 points per decade over frequencies ranging from 10 kHz to 10 mHz.

## 3. Results and Discussion

### 3.1. Single-Layer-Coated Steel

#### 3.1.1. Impedance Characteristics and Time Dependence of Corrosion

Initially, in order to characterize the corrosion protection effect of the single-layered polysiloxane-based coating formulated with active zinc phosphate pigments, the painted steel samples were only baked at 220 °C, without applying further heat treatment. The coating was applied on ideally rough, cold-rolled sand-blasted steel sheets that ensured good adhesion. The time variation of corrosion of painted steel samples was monitored with a series of measurements, in which the OCP and EIS measurements were repeated cyclically. In the early stage of immersion in a 5% NaCl solution, a 10 min waiting time was applied between recording individual impedance spectra, during which the OCP was measured. The impedance spectra in this time period were measured between 10 kHz and 10 Hz. Later, when corrosion started, and impedance decreased significantly, a larger frequency range of 10 kHz–10 mHz was used and a 30 min waiting time was applied between recording individual impedance spectra.

**Figure 3:**
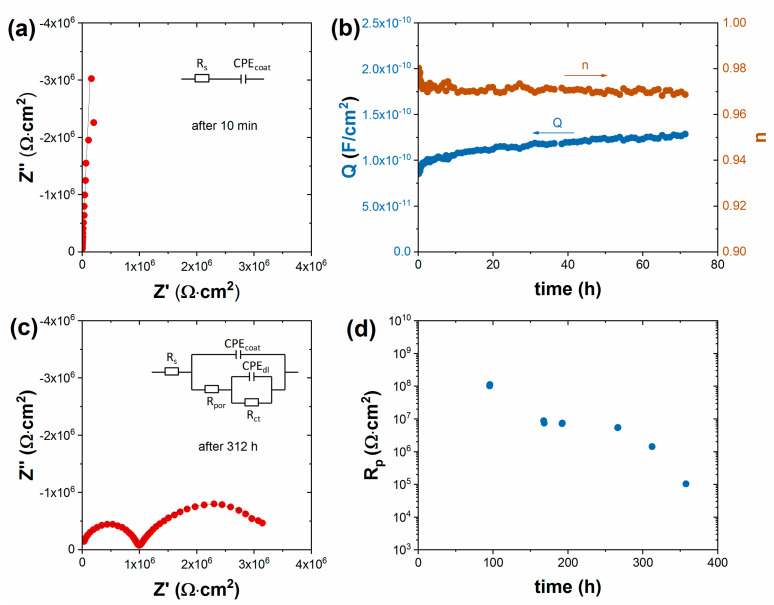
EIS results of single-layer-painted (68 μm) cold-rolled steel in 5% NaCl solution. (**a**) Impedance spectrum measured after 10 min of immersion, (**b**) variation of coating CPE values in the first 3 days of immersion, (**c**) impedance spectrum measured after 312 h of immersion, and (**d**) change in polarization resistance over time in a long time period.

Figure 3 shows the results of the impedance measurement series on single-coat-painted (68 μm) cold-rolled steel in 5% NaCl solution. In the beginning, the coating acted as a barrier with no electrochemical reaction taking place. For several days, only the frequency-dependent non-ideal capacitance of the coating could be determined from impedance (Figure 3a). Impedance spectra in this time period could be modeled with an equivalent circuit containing only the coating capacitance and solution resistance, as indicated in the inset of Figure 3a. These impedance characteristics were typical for defect-free coatings. An increase in coating capacitance took place with increasing time, which was due to the water uptake process (Figure 3b). The polarization resistance could be determined from impedance only after 4 days, which indicated the start of the corrosion processes (Figure 3d). When corrosion started, the polarization resistance decreased continuously. At the end (312 h), the spectra became two-time-constant, containing the Faraday impedance and coating elements typical for damaged coated metals (Figure 3c).

#### 3.1.2. Influence of the Layer Thickness and Steel Processing Type on Corrosion

The influence of the coating thickness was investigated in order to determine the maximum protective capacity of the coating in its basic state, without heat exposure. The corrosion-resistance properties of the coatings were tested for cold-rolled steel in two different layer thicknesses, around 70 and 100 μm. The first one is the sufficient layer thickness, and the second one is the recommended thickness for the coating system. For hot-rolled steel, the thinner layer thickness (70 μm) was applied to compare the steel-type effect. In the case of the following presented results, the exact determined layer thickness is indicated.

**Figure 4:**
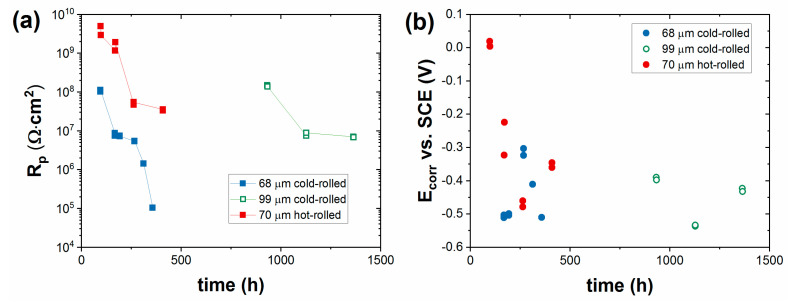
Effect of coating thickness and steel type on time dependence of (**a**) polarization resistance and (**b**) corrosion potential of single-coat-painted steels in 5% NaCl solution.

Figure 4 shows the time variation of the polarization resistance (R_p_) and the corrosion potential (E_corr_) determined on a single-layer-painted cold-rolled steel sheet with two different coating thicknesses, 68 μm and 99 μm. It can be observed that the barrier property of the coating depends significantly on the thickness of the coating. Initially, both are providing insulation for several days, but an order of magnitude difference was observed at the onset time of corrosion; the 68 μm thick coating provided insulation for 95 h, and the 99 μm thick coating provided insulation for 930 h. When corrosion started at the defect points of the coating, the R_p_ values began to decrease continuously over time. The corrosion potential values also indicated an active iron dissolution process. Salt-spray chamber tests (Table 1) confirmed the results of impedance, that the thicker paint (104 μm) layer provided much better corrosion resistance.

The method of steel sheet processing (cold-rolled, hot-rolled) can also play a role in the corrosion resistance of heat-resistant paint coatings. Especially in the case of cold-rolled steels, oxide formation can be significant at higher temperatures, which can result in coating adhesion problems. Therefore, we also examined the effect of the processing method of the base metal on the corrosion-resistance properties. It was revealed that the type of steel substrate (cold-rolled and hot-rolled) had no significant influence on the anticorrosion behavior of coating (Figure 4). The onset of corrosion was practically the same for the two types of steel, 95 h and 97 h. This was consistent with the test results of the salt-spray chamber (Table 1), where only small differences were observed between the exposure times referring to the unchanged condition of painted cold-rolled and hot-rolled steel sheets. In all cases, the corrosion took place only at a few defect points of the panels after the salt-spray chamber test.

#### 3.1.3. Effect of the Heat Treatment at 300 °C on Corrosion of Single-Coated Steel Samples

**Figure 5:**
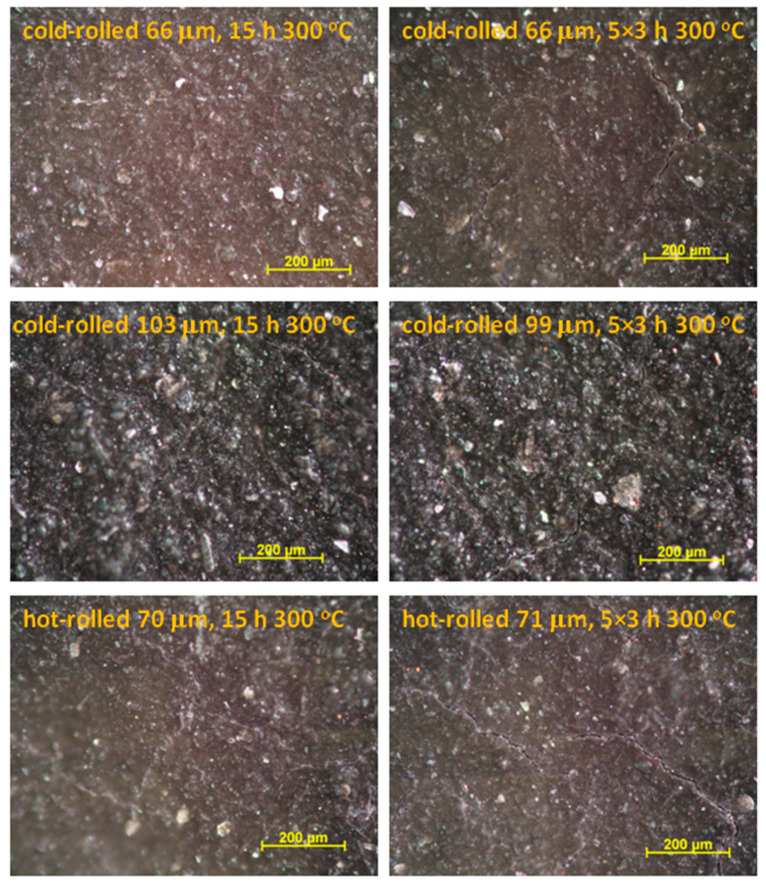
Optical microscopy images of single-layer-painted steel panels after heat treatment.

Comparative tests were performed to study the effect of heat treatment on the corrosion resistance of the coatings. Thermal equipment during operation may reach 300 °C, which is a critical temperature with respect to adhesion due to the formation of thin oxide on the steel surface [22]. For this reason, this temperature was chosen to maximize the stress on the coating. The effects of short cyclic heat load (5 × 3 h) and long-term single heat load (15 h) at 300 °C on the corrosion resistance of the coated steel sheets were compared. The cyclic method more realistically characterizes the thermal expansion conditions that may occur with a wood-heated fireplace, while the continuous method simulates a steadily operated chimney. Figure 5 shows the optical microscopic images of heat-treated panels. Microcracks were formed in the coatings in all heat-treated samples due to different thermal effects, e.g., thermal expansion. Cyclic heat treatment caused more drastic changes in morphology; the cracks were slightly wider and longer than those in the case of single continuous heat treatment. Microfractures are typically 1–5 microns wide.

**Figure 6:**
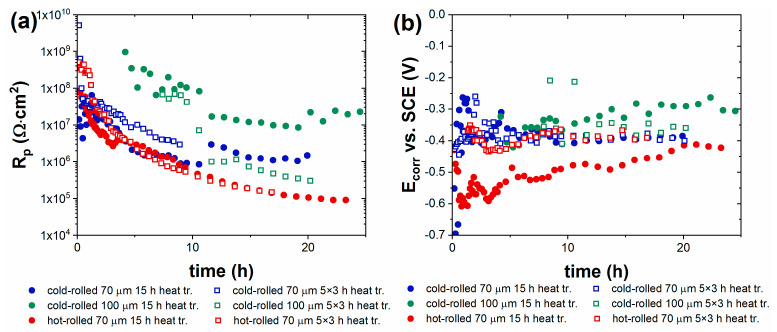
Time variation of (**a**) polarization resistance and (**b**) corrosion potential of heat-treated single-layer-painted steel panels in 5% NaCl solution.

Figure 6 shows the time variation of polarization resistance (R_p_) and corrosion potential (E_corr_) of the heat-treated painted steel samples determined by a series of electrochemical impedance spectroscopy measurements. In the case of thinner (60–70 μm) coatings, R_p_ could be determined immediately after contact with a 5% NaCl solution. The value of R_p_ was initially in the range of 10^9^ Ω·cm^2^ and decreased continuously over time. This was because the electrolyte reached the substrate steel through the microcracks in the coating, and the corrosion process could be measured by impedance measurement in the early phase of corrosion. The corrosion potential (E_corr_) values were around −400 mV vs. SCE in the beginning, which indicated that active iron dissolution was taking place. A slight difference between the R_p_ values was obtained depending on the type of heat treatment and the type of steel substrate, but the difference was not significant.

Thicker (100 μm) coatings had significantly better corrosion resistance despite the microcracks observed on the surface (Figure 5). The coating acted as a barrier during the initial 4–6 h, and the corrosion, to a measurable extent, started only after this period (Figure 6). The reason for this was that the depth of microcracks is assumed to be smaller than the thickness of the coating; therefore, initially, the electrolyte did not even reach the steel substrate.

The salt-spray chamber tests also gave similar results; the heat-treated 100-micron-thick coatings withstood salt-spray exposure an order of magnitude longer than the heat-treated 60-micron-thick coatings (Table 2). Both the salt-spray chamber test and impedance results proved that it is necessary to apply a thickness of 100 microns for the application of the investigated one-layer paint coating.

### 3.2. Bilayer Coating System Containing Zinc-Rich Primer and a Barrier Topcoat (Bilayered Coating I)

**Figure 7:**
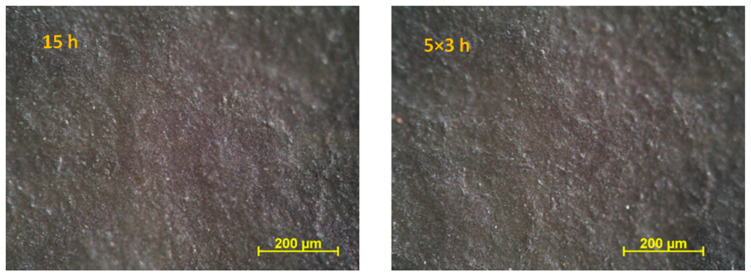
Optical microscopy images of heat-treated Bilayered Coating I system containing zinc powder primer and a barrier topcoat.

A two-layer coating system consisting of a primer containing ethyl silicate binder and zinc powder and a barrier topcoat is often recommended for fireplaces due to its high heat resistance. Therefore, we also examined the corrosion protection effect of this type of coating system, which served as a reference. The coatings were applied to a sand-blasted cold-rolled steel surface with a thickness of 120 ± 5 μm. Figure 7 shows the optical microscopic images of bilayer-painted cold-rolled steel sheets exposed to heat treatment. As can be seen in the images, the surface is continuous; microcracks did not appear, neither as a result of short cyclic heat load (5 × 3 h) nor under long-term single heat load (15 h) at 300 °C.

**Figure 8:**
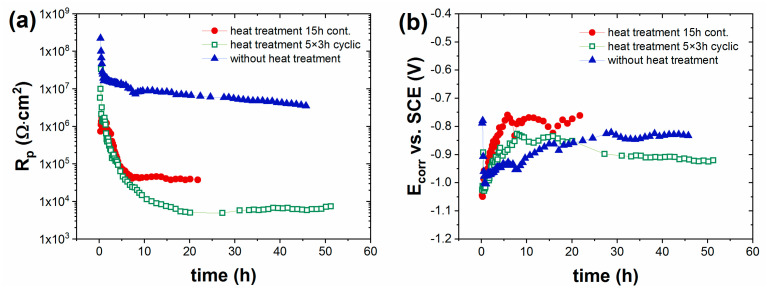
Time variation of (**a**) polarization resistance and (**b**) corrosion potential of Bilayered Coating I-coated cold-rolled steel in 5% NaCl solution. Thickness of coatings: 120 ± 5 μm.

**Figure 9:**
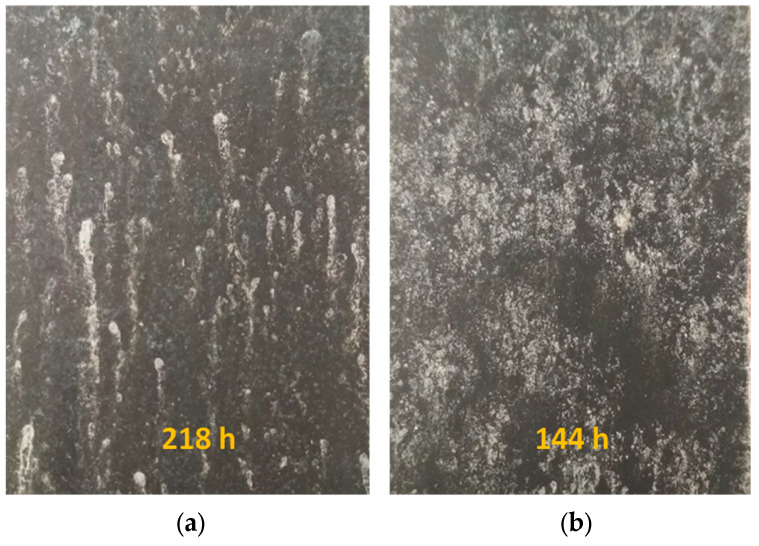
Pictures of heat-treated bilayer-coated steel sheets after salt-spray test. (**a**) 15 h at 300 °C; (**b**) 3 × 5 h at 300 °C.

The corrosion-resistance properties of the coating were studied by EIS and salt-spray chamber tests. Figure 8 shows the time variation of the polarization resistance (R_p_) and corrosion potential (E_corr_) of the heat-treated and non-heat-treated painted steel samples in 5% NaCl, determined by a series of electrochemical impedance spectroscopic measurements. It was found that corrosion could always be measured directly after contact with a 5% NaCl electrolyte, and the R_p_ values decreased drastically over time. There were orders of magnitude differences in the R_p_ values of heat-treated and non-heat-treated painted steel sheets. The R_p_ value of the panel without heat treatment was 7 × 10^6^ Ω·cm^2^, while the R_p_ values of heat-treated samples were 4 × 10^4^ Ω·cm^2^ (15 h at 300 °C) and 5 × 10^3^ Ω·cm^2^ (5 × 3 h at 300 °C) after 20 h in 5% NaCl solution. The very negative values of corrosion potential, varying between −1.1 V and −0.8 V, indicate that zinc dissolution was the dominant anodic process [11,12]. The photographs shown in Figure 9, taken after the salt-spray test, also confirm that the formation of white rust was dominant on the entire painted surface.

Salt-spray chamber tests (Table 3) confirmed the EIS results: cyclic heat treatment caused more drastic corrosion than longer, single heat treatments. At the beginning of corrosion, zinc dissolution was dominant, which was also shown by the appearance of white rust. A few red rust spots (iron dissolution) also appeared on heat-treated samples at higher exposure times.

In summary, the bilayer coating system containing zinc powder primer and a barrier topcoat had excellent heat resistance, with no microcrack formation in the coating, as can be seen in the optical micrographs. However, its corrosion resistance was much worse than that of the single-layer coating based on polysiloxane binder and Zn phosphate pigment. In addition, white rust formed during corrosion, and it appeared very quickly on the surface of the coating; thus, even though the zinc powder cathodically protects the surface of the substrate steel from corrosion, it is not suitable for grill coatings due to aesthetic considerations.

### 3.3. Ambient Curing Coating System Consisting of Zinc Phosphate Primer and a Barrier Topcoat (Bilayered Coating II)

**Figure 10:**
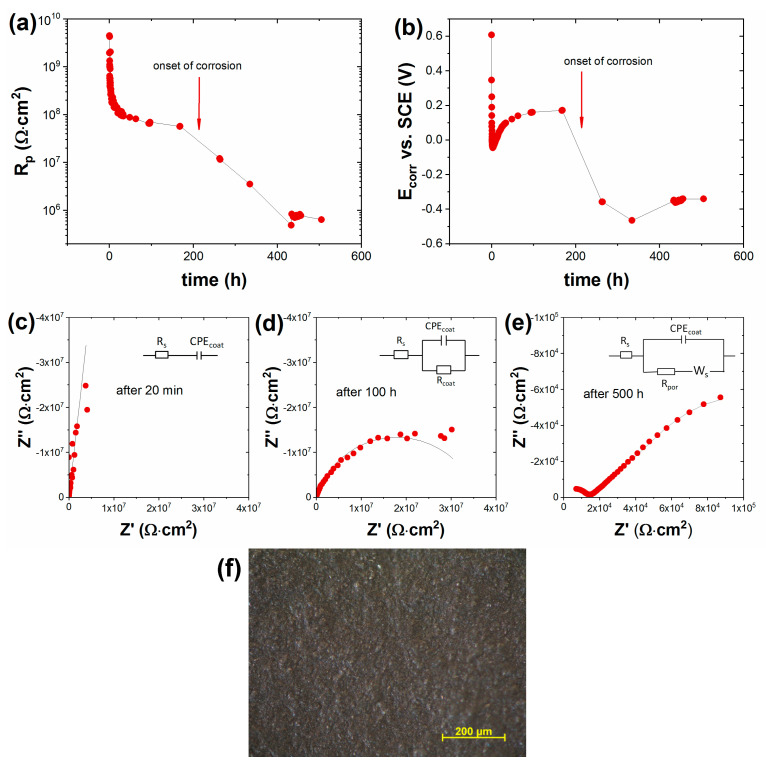
Time dependence of (**a**) polarization resistance and (**b**) corrosion potential of heat-treated (17 h at 300 °C) Bilayered Coating II-painted (48 μm primer + 20 μm top) sand-blasted cold-rolled steel in 5% NaCl solution. (**c**–**e**) Characteristic impedance plots measured after different times with corresponding equivalent circuit models in the inset. (**f**) Optical micrograph of the coated heat-treated steel surface before corrosion test.

The corrosion protection effect of another innovative approach, an ambient–cured coating system consisting of active zinc phosphate-containing primer and a barrier topcoat, was also investigated. Figure 10 shows a typical example of the time dependence of the anticorrosion behavior of this type of bilayer coating system. The coating was applied on sand-blasted cold-rolled steel, and panels were heat-treated at 300 °C for 17 h.

Initially, in the first few minutes, the coating had insulating behavior; no significant electrochemical process took place. The polarization resistance decreased continuously over time (Figure 10a) and then reached an almost constant value; a plateau can be observed near R_p_: ~60–80 MΩ·cm^2^. At the same time, the corrosion potential (Figure 10b) first decreased to −0.1 V and then increased again, and it reached a plateau around E_corr_: ~0.2 V. Presumably, in this time period, the NaCl electrolyte penetrated through the pores of the coating and the surface of the steel became accessible. Based on the potential change, it can be assumed that the active zinc phosphate pigments of the primer were dissolved due to contact with the electrolyte and provided active anodic protection to the steel surface [16], protecting it against the onset of corrosion for days. Corrosion only appeared after approximately 200 h, which was also indicated by a significant decrease in the polarization resistance and corrosion potential values. After the corrosion started, R_p_ decreased by two orders of magnitude, and E_corr_ fell to −0.4 V, which is a value characteristic of active iron dissolution.

The above-described time dependence of corrosion behaviour is well illustrated by the shape of impedance spectra shown in Figure 10c–e. Initially, in the first few minutes, only the coating capacitance and solution resistance characterized the system (Figure 10c); the coating resistance could not be determined, or could only be determined with a large error. After 1 h, the coating resistance became measurable, and the impedance spectra between 1 and 200 h could be fitted with a one-time-constant equivalent circuit model R_s_(CPE_coat_|R_coat_) as shown in Figure 10d. After the start of corrosion, the second time-constant characteristic of the Faraday reaction of corrosion appeared in the spectra, which was a diffusion-controlled process, and could be fitted with good accuracy with a finite length diffusion element (Figure 10e).

Bilayered Coating II showed better corrosion resistance in heat-treated samples than the above-presented single-layer coating, where, as can be seen in Figure 6, the corrosion process started after a few minutes, or after 4–6 h in the case of a 100 μm coating. This is a consequence of the fact that the surface of the coating is coherent; no microcracking developed as a result of heat treatment (Figure 10f). Thus, its heat-resistance properties proved to be better than those of the single-layer coatings.

### 3.4. Effect of the Surface Roughness on the Anticorrosive Properties of Coatings

Previously presented results were all obtained on coatings applied on ideally rough, sand-blasted steel sheets that ensure good adhesion. In cases of using thin sheet steel for manufacturing outdoor grill equipment, steel panels cannot be sand-blasted without deformation. Therefore, the impact of the surface preparation of the substrate on the anticorrosive properties of different coatings was also an important question. In this work stage, the anticorrosive properties of the investigated coating systems were compared on sand-blasted and smooth, non-sand-blasted substrate steel surfaces.

**Figure 11:**
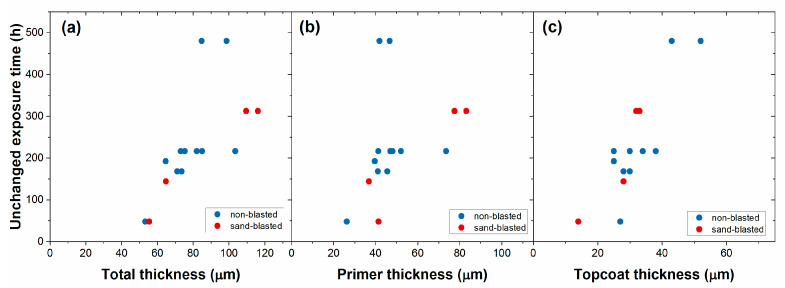
The effect of the (**a**) total, (**b**) primer, and (**c**) topcoat thickness on the unchanged exposure time in the salt-spray chamber. The panels were heat-treated (300 °C, 17 h), unroughened or sand-blasted cold-rolled steel sheets, coated with Bilayered Coating II.

Figure 11 shows the summary of salt-spray chamber corrosion tests that were performed on unroughened and sand-blasted steel panels coated with the ambient-cured coating system containing zinc phosphate active pigment (Bilayered Coating II). A strong correlation between coating thickness and unchanged exposure time was observed. It was found that the thickness of both layers, the primer and topcoat, also plays a role in corrosion resistance. Furthermore, it was found that the coating had the same good corrosion-resistance properties on the unroughened surface as on the sand-blasted steel surface. The corrosion resistance of the coating was not sensitive to the method of surface preparation.

**Figure 12:**
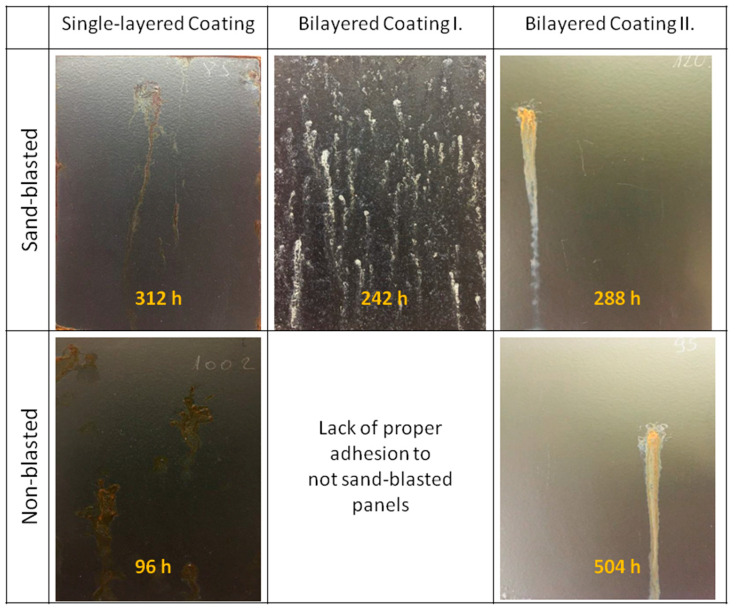
Pictures of the three investigated coating systems after salt-spray test. Before salt-spray tests, all panels were exposed to continuous heat treatment at 300 °C for 15–17 h.

Figure 12 shows the photographs of three different coating systems after heat treatment at 300 °C and the salt-spray chamber test. The three coating systems were also applied on unroughened and sand-blasted steel. The coating containing the zinc powder primer and top layer could not be formed on an unroughened surface, due to poor adhesion. In the case of a single-layer coating, the coating could be formed on a non-blasted steel surface with proper adhesion, but its corrosion resistance was worse than that of the coating formed on steel with a sand-blasted surface. In the case of the bilayer coating containing zinc phosphate pigment, both unroughened and sand-blasted steel gave similar, excellent corrosion resistance results. For this reason, this coating system promises to be suitable for heat-resistant painting of thin steel sheets.

**Figure 13:**
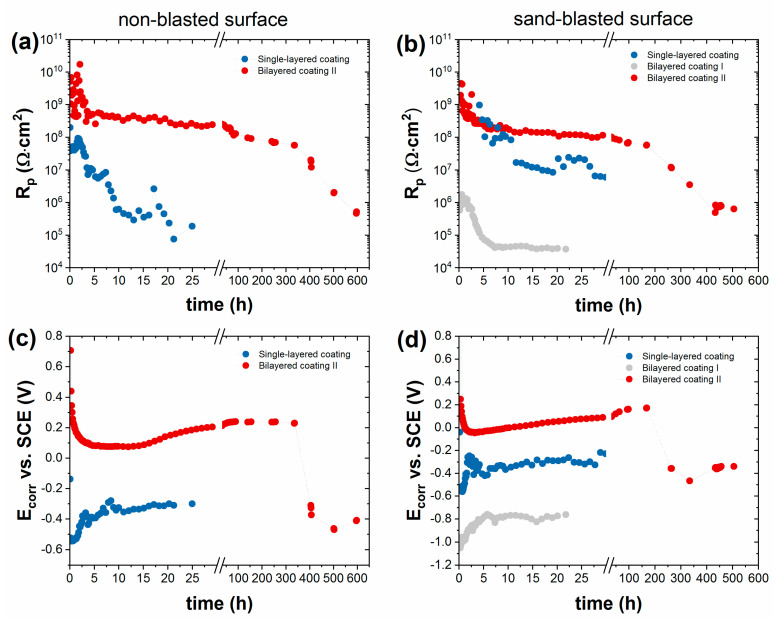
Influence of the surface preparation method on the time dependence of polarization resistance and corrosion potential of heat-treated (15 h–17 h at 300 °C), painted cold-rolled steel in 5% NaCl solution. (**a**) Polarization resistance and (**c**) corrosion potential of coatings applied on non-blasted substrates. (**b**) Polarization resistance and (**d**) corrosion potential values on coatings on sand-blasted substrates.

Figure 13 compares the impedance spectroscopy results obtained on the corrosion behavior of three different types of coatings on unroughened and sand-blasted steel surfaces. Each panel was exposed to heat treatment at 300 °C for 15–17 h in continuous mode. The figures show the changes in polarization resistance and corrosion potential over time during the entire corrosion test. In the case of Bilayered Coating II containing zinc phosphate active pigment, the time dependence of the polarization resistance was similar in the case of both the sand-blasted and the non-blasted steel surfaces. Zinc phosphate provided anodic protection to the steel surface for several weeks, where the polarization resistance was almost constant, 60–80 MΩ·cm^2^, and, at the same time, the corrosion potential also reached a nearly constant value of 0.2 V. Corrosion started only after 200 h for the sand-blasted samples and 400 h for the polished ones. The single-layer coating containing zinc phosphate active pigment formed on the sand-blasted steel surface acted as an insulator for several hours, and only after this time could the corrosion potential and polarization resistance be measured. When this happened, rapid deterioration began as the microcracks formed in the coating, functioning as active corrosion sites. Nevertheless, in a shorter period of time (first 10 h), the single-layer coating exceeded the corrosion resistance of the two-layer coating (Figure 13). On the other hand, the corrosion damage was much faster on the non-blasted surface, starting after a few minutes. The Bilayered Coating I containing the zinc powder primer provided significantly weaker corrosion resistance than the other coatings. The negative value of the corrosion potential was typical for active zinc dissolution, which was significant from the beginning of the exposure.

## 4. Conclusions

In this study, the corrosion protection effects of three different heat-resistant paint coating systems were studied. Two of them were novel, innovative approaches: a direct-to-metal single-layer polysiloxane-based oven-dried coating and an ambient-cured bilayer coating system—both were developed at Industrial Paint Research Ltd. The third coating was a traditional heat-resistant zinc-rich primer-based silicate bilayer coating. The influence of the layer thickness, heat treatment, steel type and surface preparation method were investigated. The following conclusions could be drawn:

The single-layer polysiloxane-based (Single-Layer Coating) heat-resistant paint:

- The corrosion resistance of Single-Layer Coating was significantly more favorable than the reference zinc powder containing two-layer paint coating (Bilayer Coating I) usually recommended for fireplaces. In 5% NaCl solution, corrosion occurs only after several weeks.
- Increasing coating thickness had a positive effect on long-term corrosion resistance.
- As a result of heat treatment at 300 °C, microcracks may appear in the coatings, which significantly affect the corrosion behavior. Thinner layers are more susceptible to cracking and corrosion.
- The 100 μm thick coating proved to be a more significant barrier, the number and width of microcracks being much smaller, and therefore it can be concluded that this type of coating system requires a thickness of 100 μm.
- The difference in corrosion resistance between the coated hot-rolled and cold-rolled steel substrates is not statistically significant.

An ambient-cured coating system (Bilayered Coating II) consisting of a zinc phosphate-containing primer and a barrier topcoat:

- Bilayered Coating II showed better heat-resistance properties than the single-layer coatings; the coating was coherent after heat treatment at 300 °C for 17 h, and no microcracking developed.
- Its corrosion resistance proved to be the best among the three tested coating systems.
- A correlation was observed between coating thickness and corrosion resistance.
- The coating resulted in excellent corrosion resistance even on unroughened, thin plates.

In summary, we can conclude that the Bilayered Coating II, which does not require high-temperature baking, has excellent corrosion resistance even on a steel surface without sand-blasting pretreatment. It proves to be a cost-saving eco-efficient painting method, even though it requires the application procedure of two layers. The single-layer coating also has excellent corrosion resistance under certain conditions and has the advantage of faster layer application.

## Figures and Tables

**Table 1:** Summary of the EIS and salt-spray chamber test results for cold-rolled steel panels painted with single-layered polysiloxane-based coating formulated with active zinc phosphate pigment.

Panel	EIS	Salt-Spray Test		
	Corrosion Starts	Total Exposure	Unchanged Exposure	Visual Note
cold-rolled 63 μm	95 h	600 h	586 h	Well-defined local corrosion points
cold-rolled 104 μm	930 h	1896 h	1104 h	Perfect surface after 288 h
hot-rolled 64 μm	97 h	600 h	360 h	144 h: 1 point red rust 260 h: H1 (s2)

**Table 2:** Summary of the salt-spray chamber test results for heat-treated (300 °C) single-layer-painted steel panels.

Panel	Cold-Rolled		Cold-Rolled		Hot-Rolled	
Thickness	62 μm	61 μm	104 μm	101 μm	55 μm	60 μm
Heat treatment	15 h	5 × 3 h	15 h	5 × 3 h	15 h	5 × 3 h
Total exposure	96 h	600 h	912 h	1344 h	96 h	96 h
Unchanged exposure	48 h	96 h	600 h	1104 h	72 h	72 h

**Table 3:** Summary of the EIS and salt-spray chamber test results for bilayer coating system containing zinc powder primer and a barrier topcoat on cold-rolled steel panels with and without heat treatment. Thickness of coatings: 117 ± 3 μm.

Panel	EIS	Salt-Spray Test		
	Corrosion Starts	Total Exposure	Unchanged Exposure	Visual Note
No heat treatment	10 min	504 h	358 h	288 h white rust 358 h white rust (no red rust)
15 h at 300 °C	10 min	240 h	218 h	72 h white rust 218 h red rust appears
5 × 3 h at 300 °C	10 min	168 h	144 h	48 h white rust 144 h red rust

## Data Availability

The raw data presented in this study are available on request from the authors.
